# Drivers of the changing abundance of European birds at two spatial scales

**DOI:** 10.1098/rstb.2022.0198

**Published:** 2023-07-17

**Authors:** Richard D. Gregory, Mark A. Eaton, Ian J. Burfield, Philip V. Grice, Christine Howard, Alena Klvaňová, David Noble, Eva Šilarová, Anna Staneva, Philip A. Stephens, Stephen G. Willis, Ian D. Woodward, Fiona Burns

**Affiliations:** ^1^ RSPB Centre for Conservation Science, Sandy, Befordshire SG19 2DL, UK; ^2^ Centre for Biodiversity and Environment Research, Department of Genetics, Evolution and Environment, University College London, London WC1E 6BT, UK; ^3^ Rare Breeding Bird Panel, Alnwick NE66 1EL, UK; ^4^ BirdLife International, Cambridge, Cambridgeshire CB2 3QZ, UK; ^5^ Chief Scientist Directorate, Natural England, Peterborough PE2 8YY, UK; ^6^ Conservation Ecology Group, Department of Biosciences, Durham University, South Road, Durham, County Durham DH1 3LE, UK; ^7^ Czech Society for Ornithology, 150 00 Prague 5, Czech Republic; ^8^ British Trust for Ornithology, The Nunnery, Thetford, Norfolk IP24 2PU, UK; ^9^ RSPB Centre for Conservation Science, Cambridge CB2 3QZ, UK

**Keywords:** abundance, biodiversity, biomass, climate change, European birds, land use

## Abstract

Detecting biodiversity change and identifying its causes is challenging because biodiversity is multifaceted and temporal data often contain bias. Here, we model temporal change in species' abundance and biomass by using extensive data describing the population sizes and trends of native breeding birds in the United Kingdom (UK) and the European Union (EU). In addition, we explore how species’ population trends vary with species' traits. We demonstrate significant change in the bird assemblages of the UK and EU, with substantial reductions in overall bird abundance and losses concentrated in a relatively small number of abundant and smaller sized species. By contrast, rarer and larger birds had generally fared better. Simultaneously, overall avian biomass had increased very slightly in the UK and was stable in the EU, indicating a change in community structure. Abundance trends across species were positively correlated with species’ body mass and with trends in climate suitability, and varied with species' abundance, migration strategy and niche associations linked to diet. Our work highlights how changes in biodiversity cannot be captured easily by a single number; care is required when measuring and interpreting biodiversity change given that different metrics can provide very different insights.

This article is part of the theme issue ‘Detecting and attributing the causes of biodiversity change: needs, gaps and solutions’.

## 1. Introduction

The Convention on Biological Diversity (CBD) has proposed an ambitious global plan to bring about transformational change in society's relationship with biodiversity, so that a shared vision of ‘living in harmony with nature’ can be achieved [1]. Central to that goal, and the policy process, are associated international and national biodiversity targets designed to drive positive change and to chart progress. Understanding how human activities drive biodiversity change at different spatial scales is crucial, with growing recognition that accelerating biodiversity loss may impair ecosystem functioning and the provision of vital ecosystem services to people [2–4]. The direct causes of these global changes are land and sea use change, exploitation of organisms, climate change, pollution and invasive alien species; within terrestrial and freshwater ecosystems, the key driver relative to impact is land use change, mainly land conversion for cultivation, livestock and plantation forest [2,3]. Despite the extent to which these processes are recognized, robust measurement and detection of biodiversity change, let alone attribution of change, remains challenging [5,6].

Although global assessments provide strong evidence of severe and accelerating biodiversity decline [2,3,7,8], some have questioned the strength of the underlying evidence, with some individual studies describing a more stable picture of biodiversity change [9–12]. The multifaceted nature of biodiversity and biodiversity change means we are often comparing different metrics, indices and dimensions of biodiversity [6,13]. Furthermore, the underlying monitoring datasets currently available are imperfect [5,8]. Many datasets suffer from temporal, spatial and taxonomic biases. In addition, they often lack formal sampling designs, constraining our ability to generalize and draw inference [5]. Added to these biases and constraints are the technical challenges of analysing heterogeneous data [14–16].

In this context, essential biodiversity variables (EBVs) have been proposed as a unified framework to understand how biodiversity is changing through time and space, and to help decision makers at different geopolitical scales make informed policy choices for the environment and for people [17]. EBVs are derived measurements required to study, report and manage biodiversity change, which focus on describing the status and trends in specific elements of biodiversity [17,18]. In this study, we focus on the EBV class, *species populations*, and the specific EBV, *species abundance*. In addition, we examine changes in species biomass reflecting the EBV, *species structure*.

Our knowledge of bird populations in Europe, built upon the endeavours of thousands of skilled amateur and professional scientists over many years, allows us to explore the factors driving changes in numbers and ranges of birds in detail. Here, taking advantage of two related, high-quality datasets on breeding bird assemblages in the United Kingdom (UK) and the European Union (EU), we examine changes in the total estimated number and biomass of birds over several decades. We start with field observations from structured bird counts using methods including census, line/point transects and territory mapping, to estimate the breeding numbers per species across a set of sample locations. We then model species' trends through time at national scales and at the level of the EU, and examine attribution by associating species’ trends with species' traits.

We begin by asking, simply, whether biodiversity as measured by species abundance and biomass has changed in these two bird assemblages and whether patterns of change differ at these two spatial scales. Specifically, we test the hypothesis that the total avian abundance and biomass has remained constant. We then test whether any change in abundance is associated with the abundance of the bird species, their habitat use, and aspects of their ecology and life history, such as body mass. We do not predict the relationship between species’ population trend and species' body mass since, while larger-bodied bird species have higher extinction rates and are more threatened [19,20], it does not necessarily follow that their species’ trends should be more negative (and few studies have addressed this question directly). For example, a long-term study of understorey birds in the Amazon showed that heavier species were no more likely to decline than lighter species [21]. Sullivan *et al.* [22] reported a positive but non-significant relationship between population trend and body mass in British birds. Further, Luther *et al.* [21] demonstrated that avian biomass remained stable over a 30-year period in primary forest, despite several species declining over that time span. Biomass declined strongly in modern disturbed landscapes as species groups, such as insectivores, were selectively lost as the habitat became fragmented and degraded by human activities [21]. In Europe, while the form of ecological and landscape degradation is different, driven more by intensification processes in agriculture and forestry, and by urbanization [23], we predict that biomass will have declined, consistent with previous findings [24,25]. Declines in both abundance and biomass of wild species seem particularly likely if agricultural intensification has enabled humans to sequester more of the total energy available from primary production.

We also predict that a decline in species abundance will be most marked in abundant species. This also follows previous work [24,26,27], including that by McKinney & Lockwood [28], who described a global process of ‘biotic homogenization’, in which environmental disturbances linked to human activities lead to a few species, the ‘winners’, replacing many other species, the ‘losers’, in widespread diversity change. Further, we predict that aspects of species' ecology, such as migratory tendency, will be associated with species’ trends. The complex life cycle of migrant birds, with long migration routes and a dependence on resources at different sites at different times of the year, puts them at greater risk than resident or short-distance migrant species, and we predict their trends to be more negative [26,29]. Finally, with increasing evidence to show that avian population trends are associated with climatic change, such that some species appear to have benefited from recent climatic warming and are increasing in number, while others are disadvantaged and are decreasing in number [30–32], we predict a positive relationship between species' trends and climatic suitability.

To summarize, we predict that: (i) the overall avian abundance and biomass will have fallen; (ii) any declines seen are most marked in abundant species; (iii) declines will be pronounced in specialist birds associated with intensively used habitats; and (iv) aspects of ecology, such as migration strategy and trends in climate suitability, will be associated with species’ trends. We test these predictions using two extensive datasets describing species' assemblages with near complete coverage of species abundances through time, over several decades.

To aid interpretation across species and categories, we present several metrics of change because each captures a different aspect of biodiversity change. Specifically, for species abundance, we present overall numerical change, positive and negative changes, percentage changes and the per annum rates of change in total abundance.

## 2. Methods

### (a) Population size estimates and population trends

Our analysis covered all breeding bird species native to the EU (including the UK, which was an EU Member State at the time these data were compiled) for which adequate data on population sizes and trends were available, comprising 378 out of 445 native species (86%) that breed in the EU [33]. We excluded non-native bird species because we lack equivalent and complete information on population sizes and trends for those species in UK and EU, though that information is improving all the time.

Population estimates for the UK covering 176 native breeding birds (following [34]) came from Woodward *et al.* [35]. The population estimates were commonly centred around the year 2016 (104 species), the period 2013–2017 (35 species), or tied to a specific census (37 species). Woodward *et al.* was the fourth report of the UK Avian Population Estimates Panel, which is a collaboration between statutory conservation bodies and non-governmental organizations. Population estimates for the UK have been collated approximately every seven years since 1997 and the data from the most recent report also fed into EU reporting (see below). Population estimates were taken from a number of sources where the panel provides the best estimate in cases where more than one option was available based on data quality. For scarce and rare species, population estimates generally come from the most recent dedicated species-specific surveys or from observations submitted to the UK Rare Breeding Birds Panel [36]. Some of these represent a census, as nearly all individuals of a rare species across occupied sites is known or can be estimated, though many combine a census with extrapolation based on sample data. For common and abundant species, the estimates are generally extrapolated from earlier population estimates, many derived from distance-sampling methods using population trend data from structured national breeding bird surveys. It is difficult to judge the accuracy of these estimates without perfect knowledge and we suspect estimates for rarer birds are likely more accurate than those for the commoner birds because they involve less extrapolation, and it is easier to survey rarer birds more accurately with localized, focused surveys. Note that only a subset of the population estimates from Woodward *et al.* [35] were published with accompanying confidence intervals. When absent, and where the population estimate was derived by extrapolating older estimates using annual time series from structured monitoring trends, we used the error around the annual time series values in a bootstrap procedure to repeatedly extrapolate the population estimate (*n* = 1000). We then took the 2.5% and 97.5% of these estimates as the bounds of the confidence interval around the population estimate.

Population size estimates for all 445 native breeding birds in the EU at a national level came from EU-level reporting [27,37,38]. Reference years for the population estimates are most commonly 2013/2014–2017 (341 species). National data are collated every six years as part of mandatory reporting by EU Member States to the European Commission under Article 12 of the EU Birds Directive (2009/147/EC) [39,40]. BirdLife International acts to collate and validate these data in close consultation with national experts on behalf of the European Commission. Each member state must report an estimate of the population size of each regularly occurring native breeding bird species and associated trend estimates. EU-level species’ population estimates were calculated by taking the geometric mean of the summed minimum and maximum national species' population estimates across countries and, where only a best single value was available, it was treated as the maximum and minimum. We approximated the standard error around these estimates as a sixth of the span of the maximum and minimum values, following Burns *et al.* [27]. Again, it is very difficult to judge the accuracy of these estimates without perfect knowledge, but we assume the estimates for rarer birds are probably more accurate than for the commoner birds, as described above.

Annual population time series (index values and associated standard errors) for UK birds came from variety of monitoring programmes covering different bird species’ groups and habitats (electronic supplementary material, table S1). Most time series come from two large-scale bird monitoring programmes for widespread and common species, and then from a range of additional bird monitoring schemes in the UK (electronic supplementary material, table S1). The majority of species' time series were derived from statistical trend models rather than raw counts or observations. A small number of datasets consisted of periodic counts or estimates of species abundance. For species with more than one dataset available, we gave precedence to the most robust dataset, based on the survey method with least bias and maximizing the sample size and period covered. Time series were included when they contained two or more comparable estimates of species abundance made between 1966 and the present, had broad geographical coverage across the species’ UK range, results/methodology for data collection and/or analysis were published, and start and end dates for estimates of status for each species were at least 10 years apart. Where zero counts were present, the time series was included from the year of the first positive count and 1% of the average value of the time series was added to each value in the time series of that species [41]. We would expect the structured design of the bird monitoring programmes in the UK (electronic supplementary material, table S1) to deliver modelled species' time series that are likely to be both relatively accurate and precise.

Annual population time series for EU birds (index values and associated standard errors) for 169 common native European bird species (1980–2017) are derived from the Pan-European Common Bird Monitoring Scheme (PECBMS) [42,43]. National time series, covering 26 of the 28 countries in the EU (the UK being an EU member at this time, while Croatia and Malta lacked adequate monitoring), were combined to produce a single EU-level time series per species ([42,43], https://pecbms.info/). Further details of national scheme designs and analysis are provided by Brlík *et al.* [42,43] (see https://pecbms.info/country/ and https://pecbms.info/methods/). For a species’ EU-level time series to be included, the most recent year of the time series must represent at least 50% of the species' current EU population and to reduce possible bias we chose to omit years for individual species where the species’ time series covered less than 5% of the species' EU population (*n* = 37 species and 289 species × years). Furthermore, to reduce uncertainty further, we omitted tawny pipit (*Anthus campestris*) and crested lark (*Galerida cristata*), both of which exhibit population trends that are imprecise and strongly negative [44]. We would expect the structured but variable design of the national bird monitoring programmes in the EU to deliver modelled species’ time series that are likely to be reasonably accurate and precise.

For EU bird species not covered by the PECBMS, population trends (long-term approx. 1980–2018) estimated at a national level came from the data reported under Article 12 of the Birds Directive, described above. For each species in each country, we estimated the mean trend ($\bar{T}$; log scale), mean national population estimate (${\bar{E}}_{N}$: log scale) and mean year the population estimate was made ($\bar{y}$), following Burns *et al.* [27]. Then, for each species and country, we used $\bar{T}$, ${\bar{E}}_{N}$ and $\bar{y}$, returning them to the measurement scale where necessary, to estimate the population size in each year (*i*) 1980–2017, as follows:

2.1 $${\bar{E}}_{Ni}={\bar{E}}_{N\bar{y}}.\lambda^{(i-\bar{y})};\mathrm{where}\;\lambda ={\bar{T}}^{(1/\mathrm{trend}\;\mathrm{period})}.$$

The resulting national-level time series were summed across all countries in each year to obtain an EU-level population time series for each species. We used a bootstrap approach to estimate 95% confidence intervals around each average time series. We would expect these less structured and intermittent population assessments to deliver modelled species' time series that would be less accurate and precise than those above.

### (b) Estimating change in total avian population size over time

A UK-level time series for the period 1966–2018 was selected for each UK species. Similarly, a single EU-level time series 1980–2017 was selected for each EU species using either those modelled on multiple national monitoring schemes where available (*n* = 167), or those derived from combined national trend and population estimates (*n* = 211). Time series and the species’ population estimates were analysed using the Bayesian hierarchical model of Rosenberg *et al.* [26,45], following the approach of Burns *et al.* [27]. First, smoothed species' time series were created using a Bayesian or frequentist generalized additive model for each species’ time series for the UK and the EU, respectively. Then, the Bayesian hierarchical model used the species' time series, plus additional data (on breeding habitat and migration strategy), alongside the species’ population estimate and estimate year (or range of years), in a hierarchical fashion to model species- and group-level (i.e. habitat- or migratory-group level) trends in total population size, reducing uncertain species' trends towards the mean for that habitat and migration class. This approach accounts for missing data at the start of species’ indices and incorporates uncertainty in both the annual estimates within the time series and around the population estimates [26]. The value of any initial missing years for a species in the time series is set to that of the first year with data and the variance associated with the missing values is increased by the square of the number of years since non-missing data. This means that as the number of years between a missing estimate and the closest year with data increases, the estimate has less and less influence on the model output.

### (c) Estimating trends in total avian biomass

We multiplied each species' population estimate and associated standard error in a particular year by the species’ average body mass (taken from [44]) to estimate biomass and used the Bayesian model to assess change in total biomass over time at the two geographical scales.

### (d) Covariates

Separately for UK and EU populations, species were split into four quartiles of abundance, each containing an equal number of species, based upon their UK or EU population size estimates, labelled as rare, scarce, common and abundant [24,27]. We classified each species according to their preferred breeding habitat use and trophic niche following Tobias *et al.* [44]. A species' migration strategy was defined as: (i) resident, (ii) partial migrant, (iii) short-distance migrant, and (iv) long-distance migrant, following Sanderson *et al.* [46]. We were also interested in the potential influence of climate change on species trends, given previous work linking species’ trends, both positive and negative, with climate projections and trends in climate suitability [30–32] and so we calculated ‘climate suitability trends' (CST) for each species at a UK and an EU level (electronic supplementary material, text S1).

A species’ CST is the slope of a regression of the logit of modelled mean annual climate suitability across the species' range against time [30]. Derivation of a CST requires first that species’ occurrence data are related to climate data taken from a relevant period (typically a 30–50-year mean climate preceding or relating to the period of species range data collection). Next species' distribution models (SDMs) are used to link species’ occurrence with a small set of four key climatic variables (electronic supplementary material, text S1). These SDMs are then applied to annual climate data to produce annual probabilities of occurrence, species by species, which are then regressed against time to create CST (for details see the electronic supplementary material, text S1).

### (e) Modelling correlates of species' trends

We estimated annual species’ population growth rates from the smoothed species time series (see above). We then modelled rate of change per species against a range of explanatory variables using a general linear model and a phylogenetic least-squares regression (PGLS) [47]. We excluded species whose habitat was classified as ‘marine’ from this part of the analysis because their CST values were less robust and meaningful. We also excluded a small number of range-restricted species for which no CST value was available (leaving *n* = 164 UK, *n* = 345 EU). For the PGLS, we used the avian phylogeny of Tobias *et al.* [44] and excluded a small number of species not in the phylogeny (leaving *n* = 163 UK and *n* = 336 EU). We used *λ* estimates from PGLS to assess phylogenetic signal in the model residuals and likelihood ratio tests to assess whether *λ* differed from zero or one. Continuous variables were *z*-transformed and two-way interactions were included between factorial and continuous variables where the interactions were considered biologically plausible. We assessed collinearity or association between pairs of explanatory variables (pairs of continuous variables: Pearson's correlation coefficient, pairs of nominal variables, Cramer's V, pairs with one continuous and one nominal variable: *R*^2^ values from single term linear regressions). For both the linear and the phylogenetic models, we used the dredge function (R package MuMIn, [48]) to consider all possible simplifications of the global model, removed nested models [49], and retained all models within six Akaike information criterion units of the minimum.

Global model:

$$\begin{array}{r} \log\;\mathrm{annual}\;\mathrm{rate}\;\mathrm{of}\;\mathrm{change}\;\mathrm{per}\;\mathrm{species}\\ ∼\mathrm{migration}\;\mathrm{strategy}\\ +\mathrm{habitat}+\mathrm{body}\;\mathrm{mass}+\mathrm{CST}+\mathrm{trophic}\;\mathrm{level}\\ +\mathrm{abundance}\;\mathrm{class}+\mathrm{migration}\;\mathrm{strategy}\times \mathrm{body}\;\mathrm{mass}\\ +\mathrm{migration}\;\mathrm{strategy}\times \mathrm{CST}+\mathrm{habitat}\times \mathrm{body}\;\mathrm{mass}\\ +\mathrm{habitat}\times \mathrm{CST}+\mathrm{trophic}\;\mathrm{level}\times \mathrm{body}\;\mathrm{mass}\\ +\mathrm{trophic}\;\mathrm{level}\times \mathrm{CST}. \end{array}$$

All analysis was undertaken in R version 4.1.3 [50].

## 3. Results

### (a) Changes in species abundance

**Figure 1.**
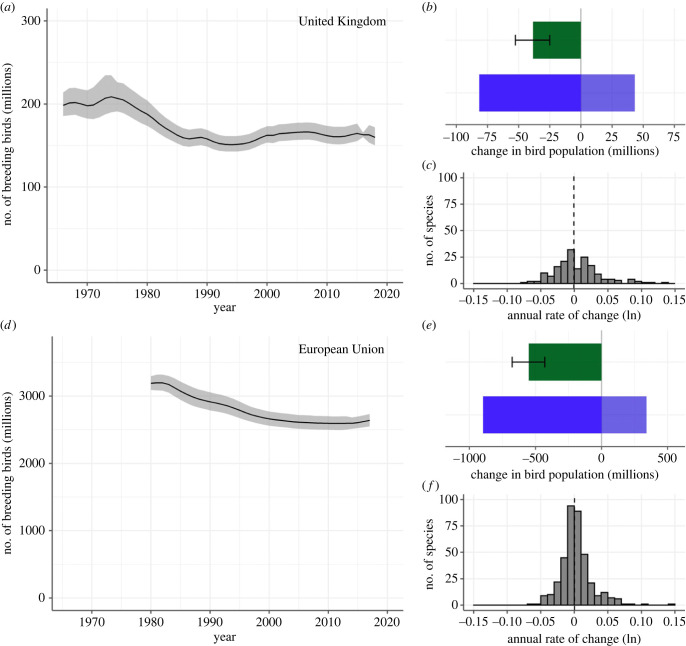
Estimated total number of breeding individuals of native breeding bird species (millions) in (*a*) the UK from 1966 to 2018, and (*d*) EU from 1980 to 2017, with shaded 95% credible intervals. Overall net change (green shading) in individuals for the UK (*b*) and EU (*e*) with 95% credible intervals (ibeam), derived from total increases among species with positive trends (pale blue) and total decreases among species with negative trends (darker blue shading). Frequency distribution of species' log-transformed average annual rate of change for species in the UK (*c*) and EU (*f*), the dashed vertical lines indicate the median value. (Online version in colour.)

We estimate the total number of native breeding birds (termed total abundance) in the UK to have declined by 38 million individuals (19%) between 1966 and 2018 (figure 1*a* and table 1), a rate of change of −0.41% per annum. Frequentist piecewise regression on a log scale indicates a change in slope around 1990 to a flatter trajectory, from a slope_1966:1992_: −1.28 (−1.47, −1.08), to a slope_1992:2018_: 0.27 (0.08, 0.45), *R*^2^ = 0.88 (numbers in parentheses are 95% credible or confidence intervals throughout). The estimated total abundance in the UK, in 2018, was 160 million breeding birds (150–172 million).

**Table 1.** Total estimated number (in millions) and estimated change in number (millions of birds, per cent, per cent per annum) of native breeding birds in the UK (1966-2018) and EU (1980-2017), showing the 95% credible interval from the Bayesian models.

geographical scale	model outputs		
	estimate	LCL	UCL
United Kingdom			
1966	198.49	185.53	213.93
2018	159.90	150.33	171.84
change	−38.49	−52.55	−24.99
per cent change	−19.44	−25.09	−13.12
per cent per annum	−0.41	−0.55	−0.27
European Union			
1980	3189.64	3092.14	3295.82
2017	2638.52	2548.81	2734.53
change	−550.46	−675.86	−429.34
per cent change	−17.27	−20.75	−13.71
per cent per annum	−0.49	−0.61	−0.39

We estimate the total abundance in the EU to have declined by 550 million individuals (17%) between 1980 and 2017 (figure 1*a* and table 1), a rate of change of −0.50% per annum. Piecewise regression on a log scale indicates a change in slope around the turn of the century, from slope_1980:2001_, −0.97 (−1.01, −0.93), to slope_2001:2017_: −0.03 (−0.10, 0.04), *R*^2^ = 0.99. The estimated total abundance in the EU in 2017 was 2.6 billion breeding birds (2.5–2.7 billion).

Total abundance decline across declining species was 82 million in the UK and 897 million in the EU, while the total increase for increasing species was 43 million in the UK and 341 million in the EU (figure 1*b,e*). The central tendency of log-transformed species' population growth rates was very close to zero in both the UK and the EU (median UK: −0.0009: median EU: 0.0002; figure 1*c,f*).

**Figure 2.**
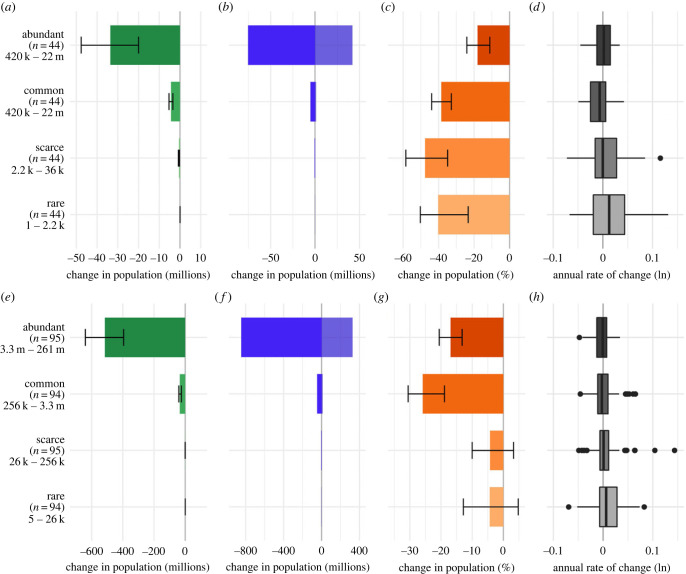
Patterns of change in native breeding bird species in the UK from 1966 to 2018 (*a–d*) and EU from 1980 to 2017 (*e–h*), disaggregated by abundance category. Net change in (*a*) UK and (*e*) EU total abundance (millions of individuals) with 95% credible intervals. Total increase in (*b*) UK and (*f*) EU species with positive trends and total decrease in species with negative trends (millions of individuals). Per cent change in total abundance (*c*) UK and (*g*) EU with 95% credible intervals. Box plot of average annual rates of change across species (*d*) UK and (*h*) EU on a log scale. In brackets, the number of species and range of species’ population sizes in each category. (Online version in colour.)

Overall, population losses were most pronounced in the most abundant categories of birds in the UK (figure 2*a*), though total proportional decline was lower in the abundant class (18%) than in the common (39%), scarce (40%) and rare (48%) classes (figure 2*c*; electronic supplementary material, table S3). By contrast, and on average, rare species had less negative population growth rates than the other abundance classes (figure 2*d*; median log-transformed population growth rates; abundant = 0.002, common = −0.007). Rare bird species in the EU also showed less negative population trajectories, on average. The median log-transformed population growth rates were weakly positive in rare (0.006) and scarce species (0.0009: figure 2*h*), whereas the average rates of change were weakly negative in common (−0.003) and abundant species (−0.0009: figure 2*h*). In contrast to the UK, the pattern of total proportional decline was reversed in the EU; rare and scarce species showed a 4% decline in total abundance, whereas there was a 26% decline in the total abundance of common species, and a 17% decline in abundant species (figure 2*f*; electronic supplementary material, table S3).

Population losses were skewed towards a small number of the most abundant bird species in the UK and EU. House sparrow (*Passer domesticus*), tree sparrow (*Passer montanus*), common starling (*Sturnus vulgaris*), willow warbler (*Phylloscopus trochilus*) and Eurasian skylark (*Alauda arvensis*) were common to both sets (electronic supplementary material, figure S1). In the UK, eight species showing the largest declines account for 76% of the decline across all 90 declining species and, in the EU, eight species showing the largest declines account for 69% of the decline across all 177 declining species. Similarly, a small number of bird species predominate in those increasing in number (electronic supplementary material, figure S1). Northern wren (*Troglodytes troglodytes*), common woodpigeon (*Columba palumbus*), Eurasian blackcap (*Sylvia atricapilla*), common chiffchaff (*Phylloscopus collybita*), European robin (*Erithacus rubecula*) and European goldfinch (*Carduelis carduelis*), are common to the increasing species sets in UK and EU. The eight most rapidly increasing species in the UK account for 77% of the increase across all 86 increasing species; in the EU, the eight species showing the largest increases account for 67% of the increase across all 201 increasing species (electronic supplementary material, figure S1).

**Figure 3.**
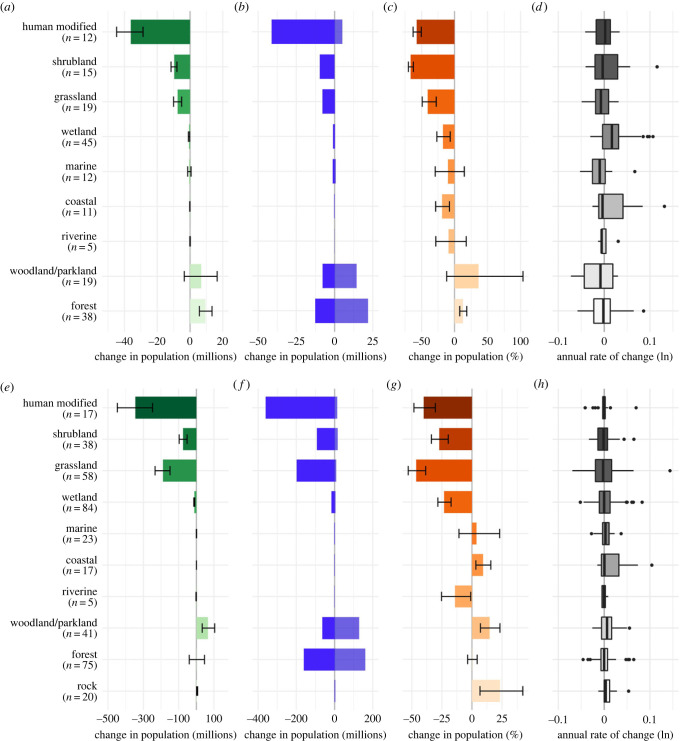
Patterns of change in native breeding bird species in the EU from 1980 to 2017 disaggregated by habitat category. Net change in total abundance in (*a*) the UK and (*e*) EU (millions of individuals) with 95% credible intervals. Total increase in species in (*b*) the UK and (*f*) EU (with positive trends and total decrease in species with negative trends (millions of individuals). Per cent change in total abundance in (*c*) the UK and (*g*) EU with 95% credible intervals. Box plot of average annual rates of change across species in (*d*) the UK and (*h*) EU on a log scale. The number of species in each habitat category is given in brackets. (Online version in colour.)

Change in total abundance also varied with the habitat use of species (figure 3; electronic supplementary material, text S1). The largest absolute declines occurred in those species associated with human-modified habitat (including intensive agriculture, urban landscapes and gardens; electronic supplementary material, table S3). Birds associated with grassland have also declined (figure 3; electronic supplementary material, table S3). Forest-associated birds in the UK were increasing to a small degree (figure 3*a–c*), but stable in the EU (figure 3*e–g*), whereas woodland/parkland-associated bird species were increasing modestly in both the UK and the EU, but with high uncertainty. Wetland breeding birds show declines in the UK (−18%) and the EU (−23%), as do riverine birds, although, in the latter case, they comprise only five species at each scale (figure 3*c,g*). Marine species abundance appeared stable in the UK and EU, while coastal species showed modest population losses in the UK (−15%) but gains in the EU (9%).

Abundance change was also associated with the migration strategy of each species. The largest numerical declines were found in resident birds and long-distance migrant birds in both the UK and EU, but the largest proportional declines were found in long-distance migrant birds in both cases (electronic supplementary material, table S3).

### (b) Correlates of species’ trends

There was limited association between the predictive variables (electronic supplementary material, table S4), except for a moderate link between body mass and trophic niche in both the UK and EU datasets, so we retained all explanatory variables in the global models. At a UK level, a single best model suggested that species' trends varied with body mass and migration strategy, with a significant interaction between these factors (electronic supplementary material, tables S5, S7, and figure S3). There was a positive relationship between species’ population trend and body mass, and species' trends were most negative in long-distance migrant birds. At an EU level, the strongest predictors of species’ trends were body mass and CST, with some influence of trophic niche, and an interaction between trophic niche and CST and abundance class (electronic supplementary material, tables S6 and S7). There was a positive relationship between species' population trend and body mass, and species’ trend and CST; the latter suggesting that climate change was a prominent driver of population change over this period, alongside other factors.

Results from phylogenetic regression suggested low phylogenetic signal in the model residuals in both the UK and the EU (maximum model UK: *λ* = 0.00 (0.00, 0.66); and EU: *λ* = 0.00 (0, 0.03)). Likelihood-ratio tests suggested there was no evidence that *λ* differed from zero, but evidence that it did differ from one. Hence, we focus on the results from the linear model but present the top models from the phylogenetic regression for information (electronic supplementary material, table S6).

### (c) Change in species' biomass

**Figure 4.**
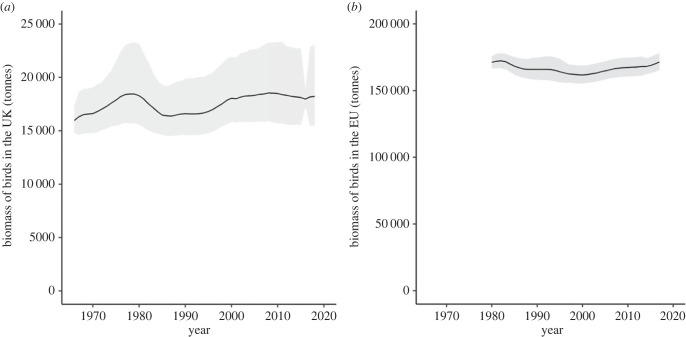
Estimated total biomass (tonnes) of individual birds of native breeding species in (*a*) the UK from 1966 to 2018, and (*b*) the EU from 1980 to 2017, with shaded 95% credible intervals.

Bird species biomass has increased but non-significantly in the UK (figure 4*a*: estimated change 2247 tonnes (−147, 6376); 14% (−1, 39) and remained stable in the EU (figure 4*b*: estimated change 322 tonnes (−6504, 7501); 0.2% (−4, 4). Change per unit area was 0.009 and 7.3 × 10^−5^ tonnes km^−^^2^ in the UK and EU, respectively. Credible intervals for UK are pinched around 2016 because it was often the reference year for population estimates, whereas the reference year(s) varied for the EU.

## 4. Discussion

Using extensive data on the breeding bird assemblages of the UK and EU, we demonstrate significant biodiversity change and loss of species abundance in the native avifauna over the last 50 years, noting that total abundance has stabilized in recent decades. We estimate a loss of around 38 million birds in the UK since 1966 and 550 million birds in the EU birds since 1980 (figure 1 and table 1). Intuitively, one might expect the average population growth rate of species in the assemblages to be negative. However, average growth rates across species were very close to zero and losses were driven by larger absolute declines in abundant species (figures 1 and 2), which tended to be smaller-bodied (see below). The most pronounced population changes were concentrated in a small number of species shaping the emergent patterns. As predicted, birds closely associated with human-modified habitats, both agricultural and urban habitats, declined strongly (figure 3).

Population trends were positively correlated with species’ body mass in the UK and EU, a pattern for which we had no *a priori* prediction since, although species' threat and extinction risk tend to be higher in larger bodied birds, that pattern can be confounded [19,20] and does not determine the relationship between species’ trend and body mass. We also show that species' trends correlate positively with climate suitability trends in the EU (electronic supplementary material, tables S3, S5 and S6), supporting previous work showing similar patterns in Europe and North America [30–32]. As predicted, species’ population trends were more negative in the more abundant species classes, and in long-distance migrant birds, but varied with niche associations in a complex way. Species' habitat use was not a strong predictor of individual species’ population trends. Population trends were, however, predicted by trophic niche, which describe a species' diet, and is perhaps closer to ecological resources that in part drive changes in species abundance. Species’ body mass emerged as a consistent predictor of species' trends in our models (electronic supplementary material, figures S3–S4 and tables S5–S6).

Avian biomass has increased slightly though non-significantly in the UK and was stable in the EU (figures 1 and 4), contrary to our expectation. Previous work, restricted to common birds in Europe, had suggested that total biomass had declined [24,25], but our analysis shows this is not the case when the entire bird assemblage is considered. The stability in total mass, despite numerical decline, suggests that species with relatively larger mass were tending to increase in number, while smaller-bodied species were tending to decline. The six species declining at both a UK and EU level, mentioned above, are all less than 80 g in mass, most considerably less; those increasing at both scales include larger birds, amongst them wood pigeon and collared dove (*Streptopelia decaocto*), both greater than 200 g (electronic supplementary material, figures S1–S2). This is a curious result, implying that total energy from primary production available to birds in Europe has remained roughly constant in recent decades, when one might expect it to have declined through increasing anthropogenic pressure. There is, however, considerable variation in the fortunes of individual species, with many falling in number but others booming (electronic supplementary material, figures S1–S2). Compensatory mechanisms have long been recognized as the basis of community stability and ecosystem resilience [51–53] and may explain the patterns we observe. Thus, bird communities in Europe are changing and presumably responding and taking advantage of changes in resource availability, which are themselves probably linked to land use and climatic change. It is unclear whether similar patterns would be seen in other regions and in other taxa.

Population declines were steepest at the start of each time series, with overall populations being stable in the UK from around 1990, and in the EU from around 2000 (figure 1). Abundance decline in UK birds (approx. 1970s–1980s) coincided with a period of rapid change in agricultural management towards more intensive methods and practices, which are thought to be responsible for the decline of specialist farmland birds [54]. Falling numbers of farmland birds have been reported since the 1970s across Europe [55], with the strongest declines reported in the north and west [56]. The similarity in patterns at the two spatial scales is to be expected given that UK data contributed to the EU (as one of 26 countries) and because EU policies applied equally over the study period. Most prominent among these is the Common Agricultural Policy, which determines how 33% [57] of the total EU budget is spent and strongly shapes agricultural practices across much of Europe; agricultural policy being known as a major driver of biodiversity loss in the EU [58].

Patterns of population change among the more abundant species in the UK and EU were quite similar, presumably shaped by the same drivers, such as land use and climate change. The same was true for rarer bird species that have fared comparatively well at both scales, a pattern that has been attributed to effective conservation planning and action in the EU [27]. Here, many rare and scarce, often larger birds, are subject to special conservation measures under the Birds Directive [40] that are translated into national conservation policies and actions (see below).

Our results parallel those of Rosenberg *et al.* [26] in North America, who modelled population change in 529 species (76% of breeding species) over a similar period, 1970–2017. They estimated a decline of 29% in breeding bird abundance since 1970, a net loss of 2.9 billion birds. As in our study, losses were highly concentrated in a small number of very abundant species including, prominently, house sparrow and common starling. Both species are considered conservation priorities in the UK in light of severe population declines [34]. The precipitous decline of the house sparrow in Europe remains something of a mystery; explanations include wide-scale changes in farming practices in the countryside that have reduced food supplies, and increased predation, shortage of food supplies and nest sites, air pollution and avian malaria in urban areas [59]. The evidence to support these hypotheses remains mixed. Starlings are predominantly grassland invertebrate feeders, often breeding in association with human habitation, and population declines in Europe are thought to be linked to changes in pastoral farming practices, including a reduction in the number of grazing cattle and loss of permanent pasture in some regions [60]. However, our understanding of their population dynamics in urban and suburban habitats is more limited [60]. Evidence is growing that bird populations within urban and suburban landscapes are, in the UK at least, in decline; that might reflect dwindling food resources with increasing urbanization, as well as the loss of nest sites for species, such as sparrow and starling, owing to house renovations [22]. Note that house sparrow and starling both feature in the top 10 declining species in North America too [26], but are invasive non-native species in that setting. Rather than being seen as conservation priorities, they are viewed as significant vertebrate pests. In that context, we need to ask whether their declines in North America are good or bad and to consider what they tell us about environmental change. Indeed, the interpretation of change and the inclusion of these species by Rosenberg *et al*. [26] was questioned [61]. Setting aside the origin of the birds, their waning populations in North America may be a valuable indicator of environmental change, driven—it is suggested—by agricultural intensification and urbanization across the continent [26,62].

Overall, the average rate of change in total abundance in North America, at −0.72% yr^−1^, is slightly higher than in the UK or EU (−0.40% and −0.52%, respectively). Curiously, the estimated loss of birds per unit area (km^2^) per year, was 3.0 and 3.4 for UK and EU, respectively, close to the figure of 2.5 for North America (estimated from Rosenberg *et al.* [26]). Tentatively, this might suggest that despite differences in biogeography and land use history, contemporary biodiversity change may be driven by similar large-scale factors, such as land use and climatic change. Note that we and Rosenberg *et al.* identify prominent declines in birds associated with intensive grassland/agricultural systems and among long-distance migrant birds. The latter showed the largest proportional declines among migration categories in the UK and EU (electronic supplementary material, table S3). Most of these European birds are Afro-Palearctic migrants wintering in savannah, deciduous and tropical forests, and coastal habitats of Africa [29]. Most migrant birds from North America winter in Central and South America in varied forests, grasslands and coastal habitats [26]. Interestingly, in North America, wetland birds were the only group to increase in number (13%), led by a 56% increase in waterfowl populations, a pattern that is not evident in our analysis. This probably reflects our classification, because ‘wetland’ incorporates waterfowl, waterbirds and wading birds. Breeding wading birds are declining strongly in Europe, whereas breeding waterbirds and waterfowl are doing comparatively well and increasing in number, many benefiting from improved site management and protection [63,64]. Both this study and Rosenberg *et al.* [26] show a slowing in the rate of decline over the last decade and there is evidence to suggest that this is driven in part by conservation actions that have acted to protect species and create and restore habitats in North America and Europe. In the EU, the Birds Directive (2009/147/EC) and the Habitats Directive (92/43/EEC) provide legal protection to listed priority species and habitats [39,40], and have been shown to benefit target bird species [65,66], leading to a considerable increase in the size of the EU's protected area network [67]. It appears that the impact of conservation actions may explain the trend towards rarer and larger species having more positive annual growth rates (figure 2), because they are targeted by those actions and may benefit most.

Standing back, the loss of abundant species in the environment may be a concern because it implies substantive changes to ecosystem structure and possibly function, and thus potential alteration to the delivery of ecosystem services [68–70]. Common species may have a high or low *per capita* influence on ecosystem services but their numerical dominance means that changes in their populations can have large implications for service provision [71]. Here, however, numerical decline in bird populations is to a degree counterbalanced by increase or stability in avian biomass, showing that bird assemblage structure is changing. Further, our analyses suggest that some of that change appears to be linked to climate suitability, and hence to climate change, alongside land use change, migration strategy and other factors.

Recent debate on global biodiversity change has revolved around the balance between ‘winners’ and ‘losers’ and on the frequency distribution of rates of change across species [9,28]. Yet an equal balance of ‘winners’ and ‘losers’ could in some circumstances result in biodiversity loss, or gain, if population changes on either side are not perfectly balanced. As we show, average population growth rates in our two datasets were close to zero. However, and overall, bird numbers have fallen because declines were pronounced in the more abundant species. This highlights the need for care in comparing and detecting biodiversity change. Leung *et al.* [11], also highlighted the possible role of ‘extreme’ population changes in driving patterns of change [14–16], and we show that a small number of abundant bird species, both increasing and declining in number, contribute strongly to the overall patterns (electronic supplementary material, figure S1).

Although our data are of relatively high quality, we are not able to generalize these results with any confidence to other taxa in this region, nor to other birds and taxa in different parts of the world. As we describe above, our estimates of relative abundance, population size and hence biomass of European birds are subject to error, only some of which we were able to capture and incorporate in our models to reflect levels of uncertainty. However, we remain confident that the major trend patterns we describe are not confounded by bias in our datasets and they align with previous work and independent data. Improvements in global, European and national species monitoring efforts are urgently needed to support similar assessments for different taxonomic groups and regions [72] and, thus, to inform conservation and policy needs.

In that respect, our ability to detect biodiversity change using these data was reasonably good but it proved challenging to attribute population change with putative drivers at the two spatial scales we explored. As Gonzalez *et al.* [73] suggest, there is a need for a more formal framework for detection and attribution of biodiversity change as this would help to harmonize research findings, improve our understanding of change and guide environmental policy.

## 5. Conclusion

Here, we demonstrate substantial biodiversity change and marked abundance loss using comprehensive data on breeding birds in Europe. Patterns of change vary with the species' body mass, their abundance, the habitat or niche they occupy, and other traits, such as trends in climate suitability. We show that large declines in total abundance conceal heterogeneity in net and gross changes in bird populations, in proportional changes and in average per annum growth rates in different groups of birds. Further, we show that avian biomass has been roughly stable in Europe, while total abundance has declined, suggesting that bird community structure is changing. We emphasize the need for care in measuring and interpreting biodiversity change, given its multifaceted nature and imperfect data.

## Data Availability

All data and model code are available in Zenodo: https://doi.org/10.5281/zenodo.7101117 [74]. The underlying European data are also available via: Eionet. 2020 Article 12 web tool: population status and trends of birds under Article 12 of the Birds Directive. Available at: https://nature-art12.eionet.europa.eu/article12/ and http://doi.org/10.5281/zenodo.4590199 [43]. Data are also provided in the electronic supplementary material [75].
